# Supplementary material for: Gene Expression Signature-Based Approach Identifies Antifungal Drug Ciclopirox As a Novel Inhibitor of HMGA2 in Colorectal Cancer
Source: Biomolecules. 2019 Nov 2;9(11):688. doi: 10.3390/biom9110688 (PMC6920845; doi:10.3390/biom9110688)
Supplement: Supplementary file 1 [file biomolecules-09-00688-s001.pdf]

Title: Gene Expression Signature-Based Approach Identifies Antifungal Drug Ciclopirox As a Novel Inhibitor of HMGA2 in Colorectal Cancer

Yu-Min Huang[1,2], Chia-Hsiung Cheng[3], Shiow-Lin Pan[4,5,6,†], Pei-Ming Yang[5,6,†], Ding-Yen Lin[5,7], Kuen-Haur Lee[5,6,8,9,*]

1 Department of Surgery, College of Medicine, Taipei Medical University, Taipei 11031, Taiwan; y.m.huang@yahoo.com.tw

2 Division of General Surgery, Department of Surgery, Taipei Medical University Hospital, Taipei 11031, Taiwan

3 Department of Biochemistry and Molecular Cell Biology, School of Medicine, College of Medicine, Taipei Medical University, Taipei 11031, Taiwan; chcheng@tmu.edu.tw

4 Ph.D. Program in Biotechnology Research and Development, College of Pharmacy, Taipei Medical University, Taipei 11031, Taiwan; slpan@tmu.edu.tw

5 Ph.D. Program for Cancer Molecular Biology and Drug Discovery, College of Medical Science and Technology, Taipei Medical University, Taipei 11031, Taiwan; yangpm@tmu.edu.tw

6 Graduate Institute of Cancer Biology and Drug Discovery, College of Medical Science and Technology, Taipei Medical University, Taipei 11031, Taiwan; khlee@tmu.edu.tw

7 Department of Biotechnology and Bioindustry Sciences, College of Bioscience and Biotechnology, National Cheng Kung University, Tainan 003107, Taiwan; lindy@mail.ncku.edu.tw

8 TMU Research Center of Cancer Translational Medicine, Taipei Medical University, Taipei 11031, Taiwan

9 Cancer Center, Wan Fang Hospital, Taipei Medical University 11696, Taipei, Taiwan

* Correspondence: khlee@tmu.edu.tw; Tel.: +886-2-26972035 (ext. 119), Fax: +886-2-66387537

† These authors contributed equally to this work.

*Corresponding author: Dr. Kuen-Haur Lee, Graduate Institute of Cancer Biology and Drug Discovery, College of Medical Science and Technology, Taipei Medical University, No. 250 Wu-Hsing Street, Taipei 11031, Taiwan. Tel: +886-2-27361661 ext. 7627, Fax: +886-2-66387537, E-mail: khlee@tmu.edu.tw

Supplementary Table S1 Top 10 chemical perturbagens with negative enrichment score for gene expression signature of overexpression of HMGA2.

Rank	Drug	Description	Enrichment score
1	Vigabatrin	A medication used to treat epilepsy, It works by inhibiting the breakdown of γ-aminobutyric acid (GABA)	-0.927
2	Viomycin	A group of nonribosomal peptide antibiotics exhibiting anti-tuberculosis properties.	-0.896
3	Nadolol	A medication used to treat high blood pressure, heart pain, and atrial fibrillation	-0.881
4	Prestwick-1082	A small-molecule perturbation from the CMAP	-0.874
5	Gly-His-Lys	A tripeptide association with α-globulin and albumin, in human blood plasma	-0.854
6	Metronidazole	An antibiotic and antiprotozoal medication	-0.853
7	Chenodeoxycholic acid	It is a bile acid. It occurs as a white crystalline substance insoluble in water but soluble in alcohol and acetic acid	-0.852
8	Prestwick-1103	A small-molecule perturbation from the CMAP	-0.828
9	Thapsigargin	The noncompetitive inhibitor of the sarco/endoplasmic reticulum Ca2+ ATPase (SERCA).	-0.825
10	Prestwick-692	A small-molecule perturbation from the CMAP	-0.801

Supplementary Table S2 Top 10 chemical perturbagens with positive enrichment scores for gene expression signature of knockdown of HMGA2.

Rank	Drug	Description	Enrichment score
1	Cloxacillin	An antibiotic useful for the treatment of a number of bacterial infections	0.834
2	Penbutolol	A medication in the class of beta blockers	0.830
3	Iopromide	A molecule used as a contrast medium	0.813
4	Pivmecillinam	An extended-spectrum penicillin antibiotic	0.808
5	Prestwick-1082	A small-molecule perturbation from the CMAP	0.794
6	Antazoline	First generation antihistamine	0.737
7	Yohimbic acid	A potent inhibitor of a human DNA Topoisomerase I and can inhibit cancer cells growth.	0.734
8	5194442	A small-molecule perturbation from the CMAP	0.712
9	Sulindac	Nonsteroidal anti-inflammatory drug (NSAID) of the arylalkanoic acid class	0.691
10	Piperine	The alkaloid and traditional medicine, a possible effect on the bioavailability of curcumin	0.684
